# Sphenopalatine ganglion: block, radiofrequency ablation and neurostimulation - a systematic review

**DOI:** 10.1186/s10194-017-0826-y

**Published:** 2017-12-28

**Authors:** Kwo Wei David Ho, Rene Przkora, Sanjeev Kumar

**Affiliations:** 1 Department of Neurology, University of Florida, PO Box 100236,1149 Newell Drive, Room L3-100, Gainesville, FL 32611 USA; 2 Department of Anesthesiology, University of Florida, 1600 SW Archer Road, PO Box 100254, Gainesville, FL 32610 USA

**Keywords:** Sphenopalatine ganglion, Block, Radiofrequency ablation, Neurostimulation, Nerve stimulation, Neuromodulation

## Abstract

**Background:**

Sphenopalatine ganglion is the largest collection of neurons in the calvarium outside of the brain. Over the past century, it has been a target for interventional treatment of head and facial pain due to its ease of access. Block, radiofrequency ablation, and neurostimulation have all been applied to treat a myriad of painful syndromes. Despite the routine use of these interventions, the literature supporting their use has not been systematically summarized. This systematic review aims to collect and summarize the level of evidence supporting the use of sphenopalatine ganglion block, radiofrequency ablation and neurostimulation.

**Methods:**

Medline, Google Scholar, and the Cochrane Central Register of Controlled Trials (CENTRAL) databases were reviewed for studies on sphenopalatine ganglion block, radiofrequency ablation and neurostimulation. Studies included in this review were compiled and analyzed for their treated medical conditions, study design, outcomes and procedural details. Studies were graded using Oxford Center for Evidence-Based Medicine for level of evidence. Based on the level of evidence, grades of recommendations are provided for each intervention and its associated medical conditions.

**Results:**

Eighty-three publications were included in this review, of which 60 were studies on sphenopalatine ganglion block, 15 were on radiofrequency ablation, and 8 were on neurostimulation. Of all the studies, 23 have evidence level above case series. Of the 23 studies, 19 were on sphenopalatine ganglion block, 1 study on radiofrequency ablation, and 3 studies on neurostimulation. The rest of the available literature was case reports and case series. The strongest evidence lies in using sphenopalatine ganglion block, radiofrequency ablation and neurostimulation for cluster headache. Sphenopalatine ganglion block also has evidence in treating trigeminal neuralgia, migraines, reducing the needs of analgesics after endoscopic sinus surgery and reducing pain associated with nasal packing removal after nasal operations.

**Conclusions:**

Overall, sphenopalatine ganglion is a promising target for treating cluster headache using blocks, radiofrequency ablation and neurostimulation. Sphenopalatine ganglion block also has some evidence supporting its use in a few other conditions. However, most of the controlled studies were small and without replications. Further controlled studies are warranted to replicate and expand on these previous findings.

**Electronic supplementary material:**

The online version of this article (10.1186/s10194-017-0826-y) contains supplementary material, which is available to authorized users.

## Review

The sphenopalatine ganglion (SPG) is also known as pterygopalatine ganglion, nasal ganglion or Meckel’s ganglion. It is the largest and most superior ganglion of sensory, sympathetic and parasympathetic nervous system. It has the largest collection of neurons in the calvarium outside of the brain. It is also the only ganglion having access to the outside environment through the nasal mucosa. SPG gives rise to greater and lesser palatine nerves, nasopalatine nerve, superior, inferior and posterior lateral nasal branches, as well as the pharyngeal branch of the maxillary nerve. There are also orbital branches reaching the lacrimal gland.

Because of its proximity to multiple important neuroanatomic structures in pain perception, SPG has been postulated to be involved in facial pain and headaches for over a century. For headache, SPG is thought to play a central role in the generation of trigeminal autonomic cephalalgia (TAC). TAC is a broad term that encompasses cluster headache, paroxysmal hemicrania, and short-lasting unilateral neuralgiform headache attack with conjunctival injection and tearing (SUNCT). It is typically distributed in the trigeminal distribution with ipsilateral cranial autonomic features. TAC is characterized by parasympathetic (lacrimation, rhinorrhea, nasal congestion and edema) activation and sympathetic dysfunction (ptosis and miosis). These clinical features can be explained by the activation of the sympathetic and parasympathetic pathways within SPG [1]. The disruption of this pathway by SPG blockade is thought to be central to relieving the headache produced by TAC. For face and neck neuralgias, connections of SPG with facial nerve, lesser occipital nerve and cutaneous cervical nerves are thought to be the mechanism [1]. Irritation of the SPG can also cause orbital, periorbital and mandibular symptoms through its connection with the ciliary and otic ganglions and reflex otalgia by its connection with the tympanic plexus. Connections of SPG with the vagus nerve may produce visceral symptoms in dysfunctional states [1]. SPG may also play an important role in vasodilation to protect the brain against ischemia from stroke or migraine with aura. This mechanism is thought to be through the postganglionic parasympathetic fibers, which are connected to the vascular beds of the cerebral hemisphere [2]. Because the upper cervical roots are connected to the superior cervical ganglion through the sympathetic trunk, which is connected to the deep petrosal nerve then to the SPG, SPG blockade is thought to be able to relieve pain from the head, face, neck and upper back [1]. This is the rationale for using SPG block in treating any head, face, neck pain refractory to conventional treatment. Through the inhibition of the sympathetic trunk, SPG block was also thought to be useful in treating generalized muscle pain including fibromyalgia and low back pain [3]. For postdural puncture headache, the pain mechanism is thought to be secondary to cerebrospinal fluid leak that exceeds the production rate, causing traction on the meninges and parasympathetic mediated reflex vasodilatation of the meningeal vessels. SPG blockade is thought to work through blocking the parasympathetic flow to the cerebral vasculature, allowing the cerebral vessels to return to normal diameter, thus relieving the headache [4].

Although the mechanism by which pain is produced from SPG is not well-characterized, SPG has been the treatment target ranging from cluster headache to low back pain. Three main types of interventions are currently available: chemical nerve block/lysis, radiofrequency ablation and neurostimulation. Some of these interventions are commonly performed in interventional pain clinics for treatment of headache resistant to conservative measures. Despite their use, the level of evidence for using SPG interventions varies widely across a myriad of conditions.

In this systematic review, we sought to systematically collect the evidence supporting the use of these SPG interventions in treating various painful conditions. We also summarized the level of evidence for each condition and intervention.

## Methods

### Protocol

This systematic review applies the guidelines issued in the latest Preferred Reporting Items for Systematic Reviews and Meta-Analysis (Additional file 1: PRISMA).

### Information sources

The electronic databases of PubMed (https://www.ncbi.nlm.nih.gov/pubmed/), Cochrane Central Register of Controlled Trials (CENTRAL, www.cochranelibrary.com), Google Scholar (https://scholar.google.com/) were searched to identify relevant articles. Additionally, references within eligible papers were screened for additional articles.

### Literature search strategy

The search was conducted in May 2017. The search strategy was based on the Population, Intervention, Comparator, Outcome (PICO) framework and was conducted to find studies on sphenopalatine ganglion block, radiofrequency ablation and neurostimulation. Population (P) was defined as patients suffering from any medical condition; intervention (I) was limited to sphenopalatine ganglion block, sphenopalatine radiofrequency ablation, and sphenopalatine ganglion neurostimulation; patients receiving interventions were compared (C) to preintervention status, patients without treatment or healthy controls; the outcome (O) needed to either qualitatively or quantitatively measure the reduction in disease severity with intervention. The complete entered search strategy in PubMed was: “(sphenopalatine) AND ganglion) AND block” for sphenopalatine ganglion block; “(sphenopalatine) AND ganglion) AND radiofrequency” for radiofrequency ablation; and (sphenopalatine AND ganglion AND neurostimulation) OR (sphenopalatine AND ganglion AND neuromodulation).

### Eligibility criteria and study selection

To be included in this review, studies had to meet the following criteria: 1. The study sample was human. 2. Interventions must be SPG block, SPG radiofrequency ablation or SPG neurostimulation. 3. Articles had to be written in English. 4. Full-Text articles had to be available. 5. Conference abstracts and reviews were excluded.

### Data items and collection

The following items were compiled in the evidence tables for SPG block (Table 2-12): first author, year of publication, medical condition treated, approach, imaging modality, medication used for the procedure, number of cases, study design and outcome. For radiofrequency ablation, the following additional items were collected: radiofrequency ablation temperature, type of radiofrequency ablation, parameter used and how to identify the correct position of the radiofrequency cannula/probe. For neurostimulation, the following additional items were collected: type of stimulator, type of stimulation and how to identify the correct position.

### Risk of bias assessment

The quality of randomized-controlled studies was assessed using the 7-item criteria in Review Manager Software version 5.35 provided by the Cochrane Collaboration [5]. The 7-item criteria contained: (1) random sequence generation; (2) allocation concealment; (3) blinding of participants and personnel; (4) blinding of outcome assessment; (5) incomplete outcome data; (6) selective reporting and (7) other bias.

### Analysis of evidence and recommendations

Level of evidence was graded based on Oxford Center for Evidence-based Medicine (1a: Systematic review of randomized-controlled trials. 1b: Individual randomized-controlled trials with narrow confidence interval. 2a: Systematic review of homogenous cohort studies. 2b: Individual cohort studies and low quality randomized-controlled trial. 3a: Systematic review of homogenous case-control studies. 3b: Individual case-control study. 4. Case series and poor-quality cohort and case-control studies. 5. Expert opinion. Grade of recommendation: A: Consistent level 1 studies. B: Consistent level 2 or 3 studies or extrapolations from level 1 studies. C: Level 4 studies or extrapolations from level 2 or 3 studies. D: Level 5 evidence or troublingly inconsistent or inconclusive studies of any level. Risk of bias in individual studies and across studies were not systematically assessed as most studies included in this review were case reports and case series.

## Results

### Overall summary

**Fig. 1:**
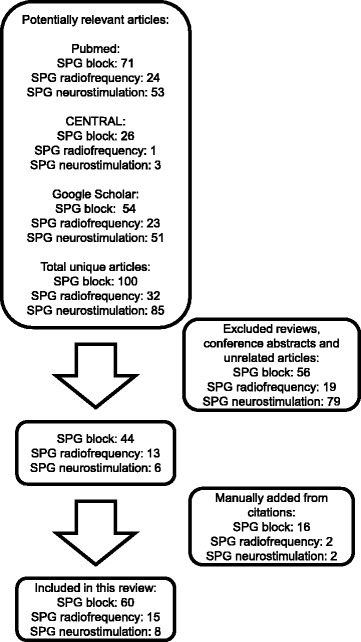
Overview of the systematic review process

The result of the search process is provided in Fig. 1. 60 articles were included for SPG block, 15 articles for SPG radiofrequency ablation, and 8 articles for SPG neurostimulation.

**Fig. 2:**
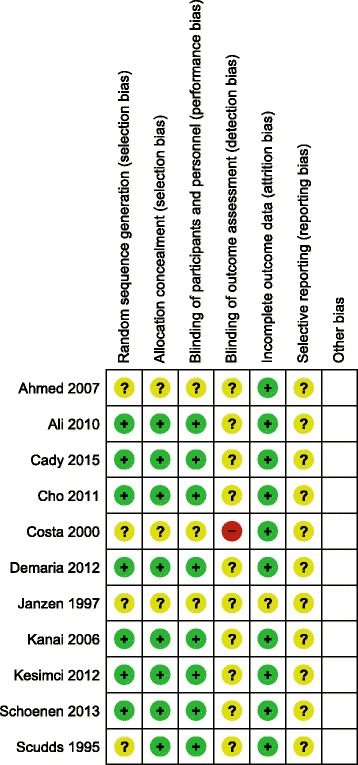
Risk of bias summary of randomized-controlled studies

The evidence levels and grades of recommendation for SPG block, radiofrequency ablation and neurostimulation are summarized in Table 1. Any study with evidence level above case series is included in Table 2. Risk of bias of randomized-controlled studies is summarized in Fig. 2. Most randomized-controlled studies included in this review have adequate randomization and blinding of participants and personnel.

**Table 1:** Summary of evidence level and grade of recommendation for SPG block, radiofrequency ablation and neurostimulation

Medical condition	Application/ Medication used in controlled studies	Number of controlled studies	Highest level of evidence	Grade of recommendation
SPG block				
Cluster headache	Cotton swab/cocaine or lidocaine	1	2b	B
Second-division trigeminal neuralgia	Lidocaine spray	1	2b	B
Reducing the needs of analgesics after endoscopic sinus surgery	Needle injection, transnasal and palatal approach/lidocaine,bupivacaine, levobupivacaine, tetracaine	6	1b	B
Reducing the pain associated with nasal packing removal after nasal operation	Needle injection, infrazygomatic approach/lidocaine	1	3b	B
Migraine	Tx360 device/ bupivicane	1	2b	B
Postdural puncture headache, sphenopalatine maxillary neuralgia, facial neuralgia, sympathetic neuralgia, post-traumatic atypical facial pain, atypical odontalgia, pain from midline granuloma, herpetic keratitis, hemifacial headache,paroxysmal hemicrania, nasal pain, hemicrania continua, trigeminal neuropathy, cancer pain, seizures associated nasal pathology, arthritic pain and muscle spasm, intercostal neuritis, persistent hiccups, ureteral colic, dysmenorrhea, peripheral painful vascular spasm, complex regional pain syndrome and hypertension	Various protocols	0	4	C
Myofascial pain	Cotton-tipped applicator, nasal spray/lidocaine	2	2b	Not recommended
SPG radiofrequency ablation				
Cluster headache	Infrazygomatic approach/80 °C, 60s ×2	0 (1 cohort study)	2b	B
Sluder’s neuralgia, posttraumatic headache, chronic head and face pain, atypical trigeminal neuralgia, atypical facial pain, chronic facial pain secondary to cavernous sinus meningioma, trigeminal neuralgia, SPG neuralgia due to herpes zoster	Various protocols	0	4	C
SPG neurostimulation				
Cluster headache	Customized to each patient, mean frequency 120.4 ± 15.5 Hz, pulse width 389.7 ± 75.4 μs, intensity 1.6 ± 0.8 mA	1	1b	B
Idiopathic facial pain, migraine	Various protocols	0	4	C

**Table 2:** Studies with evidence level above case series in SPG block, radiofrequency ablation and neurostimulation

Evidence level above case series								
Author	Year	Medical problems	Approach	Imaging	Medication	Number of cases	Study design	Outcome
SPG Block								
Berger et al. [32]	1986	Low back pain	Cotton tip applicator and transnasal needle	None	Cocaine or lidocaine	7 cases with cocaine, 7 cases with lidocaine, 7 controls	Case-control	No statistical significance between cases and controls
Slade et al. [51]	1986	Tear secretion with topical anesthesia	Needle injection, through the greater palatine foramen	None	2% lidocaine	10	Case-control (using self as control)	Tear secretion significantly reduced by 73% (*p* < 0.001)
Henneberger et al. [36]	1988	Nicotine addiction	Cotton tipped applicator, transnasal approach	None	Bupivacaine, cocaine or saline	6 with bupivacaine, 5 with cocaine, 6 with saline	Double-blind placebo-controlled	Significantly fewer symptoms of discomfort for patients in the anesthetic treatment groups than placebo group
Silverman et al. [37]	1993	Experimentally induced pain (submaximal effort tourniquet test)	Cotton tipped applicator	None	20% lidocaine and epinephrine	16 healthy volunteers	Double-blind, cross-over study	No significant difference between experimental and placebo group.
Scudds et al. [3]	1995	Chronic muscle pain syndrome	Cotton tipped applicator, transnasal approach	None	4% lidocaine	42 with fibromyalgia, 19 with myofascial pain syndrome	Double-blind randomized controlled	No statistical significance between 4% lidocaine and placebo
Janzen et al. [30]	1997	Myofascial pain syndrome and fibromyalgia	Nasal spray	None	4% lidocaine	42 with fibromyalgia, 19 with myofascial pain syndrome	Double-blind, placebo-controlled	No statistical significance between 4% lidocaine and placebo
Ferrante et al. [31]	1998	Myofascial pain syndrome of the head, neck and shoulders	NA	None	4% lidocaine	13 cases, 7 controls	Double-blind, placebo-controlled, crossover design	No statistical significance
Costa et al. [6]	2000	Cluster headache (nitroglycerin induced)	Cotton tipped applicator, transnasal approach	None	10% cocaine or 10% lidocaine	6 episodic CH, 9 chronic CH	Double-blind, placebo-controlled,	All patients with induced pain responded to cocaine after 31.3 min and lidocaine after 37 min
Hwang et al. [23]	2003	Removal of nasal packing after nasal operation	Needle injection into the greater palatine canal	None	1% lidocaine	11	Case-control	Injection side had significantly lower pain than the control side
Kanai et al. [11]	2006	Second division trigeminal neuralgia	Nasal spray	None	Lidocaine	25	Randomized control	Significantly decreased pain with intranasal lidocaine spray
Ahmed et al. [18]	2007	Sinonasal surgery intraoperative isofluorane consumption, hypotensive agents used, postoperative pain	Bilateral SPG block, injected between the middle and inferior turbinates	None	0.5% lidocaine and epinephrine.	15 cases, 15 controls	Randomized-controlled	Significantly reduced intraoperative isofluorane consumption and esmolol use, postoperative tramadol use and postoperative pain.
Ali et al. [20]	2010	Endoscopic trans-nasal resection of pituitary adenoma, anesthetic, vasodilator and analgesic sparing effect	Bilateral SPG block, injected between the middle and inferior turbinates	None	1.5% lidocaine and epinephrine	15 cases and 15 controls	Randomized-controlled	Significantly reduced in sevoflurane and nitroglycerine consumption, emergence time, postoperative pain and need of meperidine analgesia.
Cho et al. [17]	2011	Endoscopic sinus surgery postoperative analgesia efficacy	Transoral, through the greater palatine foramen	None	0.25% bupivacaine with epinephrine	60	Double-blind randomized, placebo-controlled	Pain not significantly different from control
Kesimci et al. [22]	2012	Endoscopic sinus surgery postoperative analgesia efficacy	Bilateral SPG block, injected between the middle and inferior turbinates	None	0.5% bupivacaine or 0.5% levobupivacaine	45	Double-blind randomized, placebo-controlled	Postoperative pain significantly reduced, also significantly few patients requiringadditional analgesics in the postoperative 24 h.
Demaria et al. [21]	2012	Endoscopic sinus surgery postoperative analgesia efficacy	Bilateral SPG block, palatal approach	None	2% lidocaine and 1% tetracaine	70	Double-blind randomized, placebo-controlled	Patients were discharged sooner than the control group. The block group also required less total fentanyl in the recovery room.
Cady et al. [15]	2015	Chronic migraine	Tx360	None	0.5% bupivacaine	38	Double blind, placebo control	Significantly decreased headache at 24 h
Cady et al. [16]	2015	Chronic migraine	Repetitive block (twice a week) with Tx360	None	0.5% bupivacaine	38	Double blind, placebo control	No statistical difference at 1 month and 6 months between treatment and control groups.
Schaffer et al. [34]	2015	Acute anterior or global headache	Tx360 device	None	0.5% bupivacaine	93	Randomized placebo-controlled	No statistically significant difference
Al-Qudah et al. [19]	2015	Endoscopic sinus surgery postoperative analgesia efficacy	Applied to the SPG region	None	2% lidocaine and epinephrine	60 (30 cases, 30 controls)	Double-blind, placebo controlled	Significant pain reduction in the SPG block group
Narouze et al. [38]	2009	Chronic cluster headache	Infrazygomatic approach	Fluoroscopy	NA	15	Prospective cohort	Mean attack intensity, mean attack frequency, pain disability index significant reduced at 1 year follow-up (*P* < 0.0005, *P* < 0.0003, *P* < 0.002, respectively)
SPG Neurostimulation								
Schoenen et al. [41]	2013	Cluster headache	ATI SPG stimulator positioned on the lateral-posterior maxilla medial to the zygoma. Customized, mean frequency 120.4 Hz, mean pulse width 389.7 us, mean intensity 1.6 mA	CT	–	28 cases, with 3 randomized settings.	Randomized controlled	Pain relief achieved in 67.1% of full stimulation-treated attacks compared to 7.4% of sham-treated attacks. P < 0.0001
Jurgens et al. [42]	2016	Cluster headache	Neurostimulator, described in Schoenen et al. [41]	CT	–	33 cases	Cohort study. Long-term follow-up from [41]	61% of patients were either acute responder (>50% relief from moderate or greater pain) or frequency responder (>50% in attack frequency) at 24 months
Barloese et al. [43]	2016	Cluster headache	Neurostimulator, described in Schoenen et al. [41]	CT	–	33 cases	Cohort study. Long-term follow-up from [41]	30% experienced at least 1 episode of complete attack remission (attack-free period exceeding 1 month)

In the following sections, we will summarize the level of evidence and grades of recommendations by the type of SPG interventions and associated medical conditions.

### Sphenopalatine ganglion block

Sixty articles were included for sphenopalatine ganglion block. Of the 60 studies, 11 were small randomized-controlled studies, and 1 was retrospective case-control study. The rest of the literature included case reports and case series. The type of blocking agent varied across studies, but they could be broadly put into three categories: cocaine, voltage-gated sodium channel blocker (local anesthetics), and a combination of voltage-gated sodium channel blocker and steroids. Voltage-gated sodium channel blocker is the most commonly used agent.

#### Cluster headache

There were nine articles on chronic cluster headaches collected through our literature search (see Table 3). One was a small double-blind placebo-controlled study (level 2b), six were case series and two were case reports (level 4, see Table 3). Costa et al. [6] reported a double-blind, placebo-controlled study using 15 cases of episodic and chronic cluster headaches. Cluster headache was induced with nitroglycerin, and SPG was treated with 10% solution of cocaine hydrochloride (1 ml, mean amount of application of 40-50 mg), 10% lidocaine (1 ml) or saline using a cotton swab previously immersed in these solutions. The cotton swab was placed in the region corresponding to the sphenopalatine fossa under anterior rhinoscopy. This was done in both the symptomatic and the non-symptomatic sides for 5 min. Patients treated with cocaine and lidocaine reported relief in 31.3 min in the cocaine group and 37 min for lidocaine group, compared to 59.3 min in the saline group. The side effect was mainly the unpleasant taste of lidocaine. This study was limited by its small number of participants, the acutely induced cluster headache from nitroglycerin, and its measure on only short-term outcome.

**Table 3:** Studies of SPG block for cluster headache

Cluster headache								
Author	Year	Medical problems	Approach	Imaging	Medication	Number of cases	Study design	Outcome
Devoghel et al. [52]	1981	Cluster headache	Needle injection. Supra-zygomatic approach	None	Pure alcohol	120	Case series	85.8% had complete relief
Barre et al. [7]	1982	Cluster headache	Cotton swab. Applied to sphenopalatine foramen. Self-application if responded to treatment	None	50 mg of cocaine flakes, then 10% and 5% cocaine solution	11	Case series	10 out of 11 had 50-100% abortion rate in spontaneous headache
Kittrelle et al. [8]	1985	Cluster headache	Lidocaine directly dropped into the nostrils	None	4% lidocaine	5	Case series	4 of 5 patients obtained relief of nitrate-induced cluster headaches
Costa et al. [6]	2000	Cluster headache (nitroglycerin induced)	Cotton tipped applicator, transnasal approach	None	10% cocaine or 10% lidocaine	6 episodic CH, 9 chronic CH	Double-blind, placebo-controlled,	All patients with induced pain responded to cocaine after 31.3 min and lidocaine after 37 min
Felisati et al. [9]	2006	Chronic cluster headache	Endoscopic needle injection that approaches the pterygopalatine fossa by way of the lateral nasal wall	None	Triamcinolone acetonide, 1% bupivacaine and 2% mepivacaine with adrenaline	21	Case series	11 out of 21 have improvement in symptoms
Yang et al. [53]	2006	Chronic cluster headache	Transnasal needle	Fluoroscopy	0.2% Ropivacaine and triamcinolone	1	Case report	60% pain relief
Pipolo et al. [10]	2010	Drug-resistant chronic cluster headache	Needle into the inferior portion of the sphenopalatine foramen (transnasal endoscopic technique-prasanna 1993	None	40 mg triamcinolone acetonide, 1% bupivacaine, 2% mepivacaine with adrenaline	15	Case series	55% experience complete subsidence of CH symptoms
Zarembinski et al. [54]	2014	Drug-resistant chronic cluster headache, with Jacob’s disease	Sphenopalatine ganglion block via mandibular notch, then radiofrequency oblation.	Fluoroscopy, CT	0.25% bupivacaine and 10 mg/ml dexamethasone	1	Case report	Pain significantly improved.
Kastler et al. [55]	2014	Cluster headache (14), persistent idiopathic facial pain (10), and other types of facial pain (18)	Infrazygomatic approach	CT	Absolute alcohol	14	Case series	76.5% of patients have 50% pain relief at 1 month

Other case reports/series using cocaine and local anesthetics as blocking agents generally reported good immediate outcomes for aborting acute cluster headache. One study using cocaine reported 10 out of 11 patients receiving 50-100% relief from spontaneous cluster headache [7], another study using lidocaine reported four out of five patients receiving relief from nitrate-induced cluster headache [8]. Because of the short-term relief from cocaine and lidocaine, steroid has been tried to prolong the relief provided by SPG block. In one case series, combination of triamcinolone, bupivacaine, mepivacaine and epinephrine helped improve severity and frequency of cluster headaches in 11 out of 21 patients [9]. The same cocktail helped 55% of the 15 treated patients in another case series [10].

In summary, SPG block has moderate evidence in treating cluster headache. The overall grade of recommendation is B for SPG block on cluster headache. The strongest evidence lies in aborting nitroglycerin-induced cluster headache using local application of cocaine or lidocaine with cotton swab through the transnasal approach. The side effect was mainly the unpleasant taste of lidocaine. Addition of steroid may provide longer relief, but the evidence remains weak (Grade C recommendation).

#### Trigeminal neuralgia

There were four articles on SPG block for trigeminal neuralgia through our literature search. One was a randomized-controlled study (level 2b), two were case series and one case report (level 4, see Table 4). Kanai et al. performed a randomized-controlled study with 25 participants with refractory second-division trigeminal neuralgia [11]. In this study, twenty-five patients with second-division trigeminal neuralgia were randomized to receive two sprays (0.2 ml) of either lidocaine 8% or saline placebo in the affected nostril using a metered-dose spray. The paroxysmal pain triggered by touching or moving face was assessed. Intranasal lidocaine 8% spray significantly decreased the paroxysmal pain for an average of 4.3 h. The side effects were limited to local irritation with burning, stinging or numbness of the nose and eye, and bitter taste and numbness of the throat. One case series [12] and one case report [13] reported immediate pain relief from nerve blocks with lidocaine and bupivacaine. One case series used a combination of dexamethasone and ropivacaine with the Tx360 applicator, which resulted in short-term pain relief [14]. Multiple blocks over time seemed to provide longer pain relief but it was restricted to isolated cases.

**Table 4:** Studies of SPG block for trigeminal neuralgia

Trigeminal neuralgia								
Author	Year	Medical problems	Approach	Imaging	Medication	Number of cases	Study design	Outcome
Peterson et al. [12]	1995	Trigeminal neuralgia	Cotton tip applicator	None	4% lidocaine	2	Case series	Pain free
Manahan et al. [13]	1996	Trigeminal neuralgia	NA	None	Bupivacaine	1	Case report	Pain free
Kanai et al. [11]	2006	Second division trigeminal neuralgia	Nasal spray	None	Lidocaine	25	Randomized control	Significantly decreased pain with intranasal lidocaine spray
Candido et al. [14]	2013	Trigeminal neuralgia, chronic migraine headache, post-herpetic neuralgia	Tx360 Nasal applicator, transnasal	None	0.5% ropivacaine and 2 mg dexamethasone	3	Case series	Satisfactory

In summary, the overall grade of recommendation is B for SPG block on trigeminal neuralgia. The strongest evidence lies in treating with 8% lidocaine nasal spray in the affected nostril. The analgesia is effective but temporary (4.3 h). It is well-tolerated with side effects limited to local irritations. Addition of steroid and use of the Tx360 applicator may be useful but there has not been a controlled study.

#### Migraine

There was one small double-blind, placebo-controlled study and one long-term follow-up of the same study (level 2b), two case series and one case report (level 4, see Table 5). Cady et al. [15] reported a randomized-controlled study using the Tx360 device and bupivacaine to acutely treat chronic migraines with repetitive SPG blockade. 38 subjects with chronic migraines were included in the final analysis. Participants received a series of 12 SPG blocks with either 0.3 cm^3^ of 0.5% bupivacaine or saline delivered with the Tx360® through each nostril, over a 6-week period (2 SPG blocks/week). SPG block was found to be effective in reducing the severity of migraines up to 24 h. However, repetitive blocks did not provide any statistically significant relief at 1-month or 6-month follow-ups [16]. The most common side effects were mouth numbness, lacrimation, and bad taste, but there was no statistical difference in frequency of side effects between the bupivacaine and saline groups.

**Table 5:** Studies of SPG block for migraine

Migraine								
Author	Year	Medical problems	Approach	Imaging	Medication	Number of cases	Study design	Outcome
Amster et al. [28]	1948	Migraine	Cotton tipped applicator, transnasal approach	None	Nupercaine, pontocaine, monocaine	4	Case series	Relief of pain and spasm in 90% of cases
Maizels et al. [56]	1999	Migraine with aura	Self-administered intranasal 4% lidocaine	None	4% lidocaine	1	Case report	Most headaches were successfully aborted for 15 months
Yarnitsky et al. [57]	2003	Migraine	Cotton tip applicator	None	2% lidocaine	32	Case series	Significant reduction in pain score during migraine
Cady et al. [15]	2015	Chronic migraine	Tx360	None	0.5% bupivacaine	38	Double blind, placebo control	Significantly decreased headache at 24 h
Cady et al. [16]	2015	Chronic migraine	Tx360	None	0.5% bupivacaine	38	Double blind, placebo control	No statistical difference at 1 month and 6 months between treatment and control groups.

Given the positive randomized-controlled study, grade of recommendation is B for short term treatment of chronic migraines using 0.5% bupivacaine with the Tx360 device®. It should be noted that the effect is only present for 24 h. and it is not suitable for patients seeking relief greater than 24 h.

#### Postoperative pain of the head and face

There were six randomized-controlled studies, one case-control study and one case series falling under this category (Table 6).

**Table 6:** Studies of SPG blocks for operative pain of the head and face

Operative Pain of the head and neck								
Author	Year	Medical problems	Approach	Imaging	Medication	Number of cases	Study design	Outcome
Robiony et al. [24]	1998	Skeletal transverse discrepancy of the maxilla	Transcutaneous truncal anesthesia of the maxillary nerve in association with transmucosal anesthesia of the sphenopalatine ganglion	None	Prilocaine carbocaine cream	12	Case series	Total anesthesia of the maxillary area facilitated the operations and appreciably reduced amount of postoperative pain
Hwang et al. [23]	2003	Removal of nasal packing after nasal operation	Needle injection into the greater palatine canal	None	1% lidocaine	11	Case-control	Injection side had significantly lower pain than the control side
Ahmed et al. [18]	2007	Endoscopic sinonasal surgery intraoperative isofluorane consumption, hypotensive agents used, postoperative pain	Bilateral SPG block, injected between the middle and inferior turbinates	None	0.5% lidocaine and epinephrine.	15 cases, 15 controls	Randomized-controlled	Significantly reduced intraoperative isofluorane consumption and esmolol use, postoperative tramadol use and postoperative pain.
Ali et al. [20]	2010	Endoscopic trans-nasal resection of pituitary adenoma, anesthetic, vasodilator and analgesic sparing effect	Bilateral SPG block, injected between the middle and inferior turbinates	None	1.5% lidocaine and epinephrine	15 cases and 15 controls	Randomized-controlled	Significantly reduced in sevoflurane and nitroglycerine consumption, emergence time, postoperative pain and need of meperidine analgesia.
Kesimci et al. [22]	2012	Endoscopic sinus surgery postoperative analgesia efficacy	Bilateral SPG block, injected between the middle and inferior turbinates	None	0.5% bupivacaine or 0.5% levobupivacaine	45	Double-blind randomized, placebo-controlled	Postoperative pain significantly reduced, also significantly few patients requiring additional analgesics in the postoperative 24 h.
Demaria et al. [21]	2012	Endoscopic sinus surgery postoperative analgesia efficacy	Bilateral SPG block, palatal approach	None	2% lidocaine and 1% tetracaine	70	Double-blind randomized, placebo-controlled	Patients were discharged sooner than the control group. The block group also required less total fentanyl in the recovery room.
Al-Qudah et al. [19]	2015	Endoscopic sinus surgery postoperative analgesia efficacy	Applied to the SPG region	None	2% lidocaine and epinephrine	60 (30 cases, 30 controls)	Double-blind, placebo controlled	Significant pain reduction in the SPG block group

Six randomized-controlled studies examined the efficacy of SPG blockade in reducing the needs of analgesics after endoscopic sinus surgery (level 2b). One study by Cho et al. [17] did not show significant difference between SPG block and placebo, but five additional randomized-controlled studies showed significant reduction in the need of post-operative analgesics in the group treated with SPG block [18–22]. The five positive studies used 0.5% lidocaine with epinephrine [18], 1.5% lidocaine with epinephrine [22], 0.5% bupivacaine or 0.5% levobupivacaine [22], 2% lidocaine and 1% tetracaine [21]. The SPG block was applied using injections, bilaterally through the transnasal or palatal approach. There was no difference in complications between the treatment and placebo group.

Hwang et al. [23] reported a case-control study to assess the efficacy of SPG block in reducing the pain associated with nasal packing removal after nasal operation (level 3b). 1% lidocaine was injected into the greater palatine canal ipsilaterally using infrazygomatic approach. Participants reported significantly lower pain on the side of the nose that received SPG block compared to the control side.

Robiony et al. [24] reported one case series (level 4) on the effectiveness of combined maxillary transcutaneous nerve block and SPG block in reducing postoperative pain for surgical correction of skeletal transverse discrepancy of the maxilla.

Given five positive double-blind placebo-controlled studies and one negative study, the grade of recommendation is B for SPG block in improving postoperative analgesia efficacy after endoscopic sinus surgery. Each study blocked SPG with injection of different local anesthetics using different approaches. In 5 studies, SPG block was consistently found to be effective in reducing the need of analgesics after endoscopic sinus surgery. A combination with maxillary transcutaneous nerve block may be also helpful but further systematic study is necessary to evaluate its efficacy. Grade of recommendation is also B for reducing pain associated with nasal packing removal after nasal surgery, using lidocaine injection through the infrazygomatic approach.

#### Head and neck cancer pain

Three case reports and series were found (level 4 evidence, Table 7). One study was SPG block and two on SPG neurolysis with phenol. The largest case series was by Varghese et al. [25], who reported 22 cases of successful treatment with 6% phenol used via nasal endoscopy, as a neurolytic sphenopalatine ganglion block, for pain caused by advanced head and neck cancer. The overall grade of recommendation is C for any of these painful conditions.

**Table 7:** Studies of SPG block for cancer pain

Head and neck cancer pain								
Author	Year	Medical problems	Approach	Imaging	Medication	Number of cases	Study design	Outcome
Prasanna et al. [58]	1993	Pain from carcinoma of the tongue and floor of the mouth	Nasal sinuscope	None	0.25% bupivacaine	10	Case series	Immediate pain relief
Varghese et al. [25]	2001	Pain due to advanced head and neck cancer	Endoscopic needle injection	None	6% phenol	22	Case series	17 out of 22 patients had significant pain relief
Varghese el al. [59]	2002	Pain due to advanced head and neck cancer	Transnasal through the sphenopalatine foramen	None	6% phenol	1	Case report	Significant pain relief

#### Postherpetic neuralgia

A total of three case reports and series were found through our search process (level 4 evidence, Table 8). All three articles reported successful treatment of postherpetic neuralgia with SPG block using local anesthetics. One study reported successful treatment of postherpetic neuralgia involving the ophthalmic division of the trigeminal nerve, by SPG block under direct visualization through nasal endoscopy [26]. Another article reported success in treating sinus arrest in postherpetic neuralgia by SPG block through trans-nasal approach utilizing cotton tipped applicators [27], and one study reported successful treatment of herpes zoster within a heterogeneous case series [28]. The overall grade of recommendation is C.

**Table 8:** Studies of SPG block on postherpetic neuralgia

Postherpetic neuralgia								
Author	Year	Medical problems	Approach	Imaging	Medication	Number of cases	Study design	Outcome
Prasanna et al. [26]	1993	Postherpetic neuralgia involving the ophthalmic division of the trigeminal nerve	Combination of stellate ganglion and sphenopalatine ganglion block, cotton tip applicator	None	Lidocaine and bupivacaine	1	Case report	Pain free
Saberski et al. [27]	1999	Sinus arrest in postherpetic neuralgia	Cotton tipped applicator, transnasal approach	None	20% lidocaine	1	Case report	No paroxysmal pain or sinus pauses immediately after block
Amster et al. [28]	1948	Herpes zoster	Cotton tipped applicator, transnasal approach	None	Nupercaine, pontocaine, monocaine	3	Case series	Relief of pain and spasm in 90% of cases

#### Musculoskeletal pain

There were two negative randomized-controlled study on head, neck and shoulder myofascial pain. There were also a small case-control study on low back pain, a small randomized-controlled study on chronic muscle pain syndrome and two large case series in our literature search (Table 9).

**Table 9:** Studies of SPG block for musculoskeletal pain

Musculoskeletal pain								
Author	Year	Medical problems	Approach	Imaging	Medication	Number of cases	Study design	Outcome
Amster et al. [28]	1948	Lumbosacral and sacroiliac pain	Cotton tipped applicator, transnasal approach	None	Nupercaine, pontocaine, monocaine	61	Case series	Relief of pain and spasm in 90% of cases
Ruskin et al. [29]	1946	Lumbo-sacral spasm	Unknown	None	Cocaine, novocaine or nupercaine	36	Case series	Pain partially or completely relieved with SPGB and intramuscular injections of ironyl and calcium ascorbate
Berger et al. [32]	1986	Low back pain	Cotton tip applicator and transnasal needle	None	Cocaine or lidocaine	7 cases with cocaine, 7 cases with lidocaine, 7 controls	Case-control	No statistical significance between cases and controls
Scudds et al. [3]	1995	Chronic muscle pain syndrome	Cotton tipped applicator, transnasal approach	None	4% lidocaine	42 with fibromyalgia, 19 with myofascial pain syndrome	Double-blind randomized controlled	No statistical significance between 4% lidocaine and placebo
Janzen et al. [30]	1997	Myofascial pain syndrome and fibromyalgia	Nasal spray	None	4% lidocaine	42 with fibromyalgia, 19 with myofascial pain syndrome	Double-blind, placebo-controlled	No statistical significance between 4% lidocaine and placebo
Ferrante et al. [31]	1998	Myofascial pain syndrome of the head, neck and shoulders	NA	None	4% lidocaine	13 cases, 7 controls	Double-blind, placebo-controlled, crossover design	No statistical significance

Successful treatment of lumbosacral pain with SPG block was initially reported in two large case series in the 1940s [28, 29]. However, further randomized-controlled studies dismissed these findings. Scudds et al. [3] reported a randomized-controlled study applying SPG block (cotton-tipped pledgelets with 4% lidocaine) to 42 participants with fibromyalgia and 19 participants with myofascial pain syndrome. He reported no statistical difference between treatment and placebo group in pain intensity, headache frequency, sensitivity to pressure, anxiety, depression, and sleep quality. Janzen et al. [30] reported a similar randomized-controlled study by applying SPG block with lidocaine spray. Forty-two participants with fibromyalgia and 19 with myofascial pain syndrome were included in his study. He again found not difference between the treatment and placebo group. Ferrante et al. [31] reported a randomized-controlled study with 13 cases of head, neck and shoulder myofascial pain and 7 healthy controls. He also showed no significant effect with SPG block. On low back pain, Berger et al. [32] reported a case-control study with 21 patients randomized to cocaine, lidocaine and saline. He did not find significant differences in outcomes. Given the negative randomized-controlled studies, it is not recommended to use SPG block on musculoskeletal pain.

#### Postdural puncture headache

There were two case series and one case report (level 4) reporting successful treatment of postdural puncture headache (Table 10). No higher-level studies were available. Cohen et al. [33] reported the largest case series of 32 cases with postdural puncture headache. In the series, 69% of the patients treated with transnasal SPG block were saved from epidural blood patch. The overall grade of recommendation is C for SPG block on postdural puncture headache.

**Table 10:** Studies of SPG blocks for postdural puncture headache

Postdural puncture headache								
Author	Year	Medical problems	Approach	Imaging	Medication	Number of cases	Study design	Outcome
Cohen et al. [60]	2014	Postdural puncture headache	Cotton-tip applicator	None	5% lidocaine	32	Case series	69% of the patients were saved from epidural blood patch
Kent et al. [4]	2015	Postdural puncture headache	Cotton-tip applicator	None	2% lidocaine	3	Case series	1 patient had relief, 2 had to get epidural blood patch.
Cardoso et al. [61]	2017	Postdural puncture headache	Cotton-tip applicator	None	0.5% Levobupivacaine	1	Case report	Symptoms relieved by 5 min.

#### Other pain syndromes of the head and face

Pain syndromes involving the head and face not belonging to any category mentioned above are summarized in Table 11. There was a negative randomized-controlled study using Tx360 device treating acute anterior and global headache [34]. There were also multiple case reports and series on the effectiveness of SPG in controlling various types of head and facial pain. Local anesthetics and steroids have been used for SPG block, while phenol and alcohol have been used for SPG neurolysis. They have been successfully used in Sluder’s neuralgia, sphenopalatine maxillary neuralgia, facial neuralgia, sympathetic neuralgia, post-traumatic atypical facial pain, atypical odontalgia, pain from midline granuloma, herpetic keratitis, hemifacial headache, paroxysmal hemicrania, nasal pain, hemicrania continua and trigeminal neuropathy. The largest case series was provided by Rodman et al. [35], documenting 147 patients with various types of nasal pain and headache. He reported that 81.3% of the patients had pain relief after receiving SPG block with a mixture of bupivacaine and triamcinolone. Schaffer et al. [34] reported a randomized placebo-controlled study using Tx360 device to treat acute anterior or global headache. A total of 93 participants were recruited in the study, but the study showed no statistical significance between the treatment and control groups. Because of the result, we do not recommend SPG block for anterior or global headache. The overall grade of recommendation is C for other types of head and facial pain, including Sluder’s neuralgia, sphenopalatine maxillary neuralgia, facial neuralgia, sympathetic neuralgia, post-traumatic atypical facial pain, atypical odontalgia, pain from midline granuloma, herpetic keratitis, hemifacial headache, paroxysmal hemicrania, nasal pain, hemicrania continua and trigeminal neuropathy.

**Table 11:** Studies of SPG blocks for other pain syndromes of the head and face

Pain syndromes of the head and face								
Author	Year	Medical problems	Approach	Imaging	Medication	Number of cases	Study design	Outcome
Ruskin et al. [62]	1925	SP maxillary neuralgia, SP facial neuralgia, SP sympathetic neuralgia, SPG cell neuralgia	Needle injection.	None	20% Cocaine, 10% silver nitrate, 70% alcohol	7	Case series	Improvements or complete relief
Stechison et al. [63]	1994	Post-traumatic atypical facial pain syndrome	Needle injection. Transfacial transpterygomaxillary access to foramen rotundum SPG and maxillary nerve	CT	First stage: 0.5% bupivacaine, Second stage: 98% ethyl alcohol and 0.5% bupivacaine in 2:1 ratio	5	Case series	3 had alcohol neurotomy and pain free at 5, 8 and 12 months. 2 responded poorly to first stage blockade and did not have alcohol neurotomy.
Peterson et al. [12]	1995	Atypical odontalgia	Cotton tip, self-application	None	4% lidocaine	1	Case report	Pain free
Saade et al. [64]	1996	Pain from midline granuloma	Self-administered SPG block	None	Lidocaine	1	Case report	Significant pain relief
Puig et al. [65]	1998	Sluder’s neuralgia	Cotton tip applicator and transnasal needle	None	88% phenol	8	Case series	90% decrease in head and face pain for 9.5-month duration
Windsor et al. [66]	2004	Herpetic keratitis	Transnasal cotton tip applicator	None	Tetracaine, adrenalin and 10% cocaine]	1	Case report	Effect of block lasts for a month. Requires months blocks
Obah et al. [67]	2006	Hemifacial and headache	Transnasal	None	4% lidocaine	1	Case report	80% reduction in pain intensity
Cohen et al. [33]	2009	Postdural puncture headache	Cotton tip applicator	None	Lignocaine	13	Case series	11 out of 13 had immediate relief of headache
Morelli et al. [68]	2010	Paroxysmal hemicrania resistant to multiple therapies	Endoscopic needle injection into the nasal mucous membrane immediately behind and over the inferior portion of the sphenopalatine foramen and into the fossa	None	Triamcinolone acetonide, 1% bupivacaine, 2% mepivacaine with adrenalin	1	Case report	Reduction in frequency and intensity of pain
Rodman et al. [35]	2012	Nasal pain or headache	Endoscopic needle injection	None	0.5% bupivacaine and triamcinolone acetonide	147	Case series	81.3% of patients have improvement
Grant et al. [69]	2014	Tension headache in pregnant woman	Cotton tip applicator	None	4% lidocaine	1	Case report	BID block for a total of 7 blocks, pain free after
Kastler et al. [55]	2014	Cluster headache (14), persistent idiopathic facial pain (10), and other types of facial pain (18)	Infrazygomatic approach	CT	Absolute alcohol	28	Case series	85.7% of patient with persistent idiopathic facial pain and 40% of other types of facial pain had 50% pain relief at 1 month
Androulakis et al. [70]	2016	Hemicrania continua	Tx360 device	None	Repetitive 0.5% bupivacaine	1	Case report	Significant improvement in headache by 14 week
Malec-Milewska et al. [71]	2015	Trigeminal neuropathy	Zygomatic approach	Fluoroscopy	65% ethanol with lidocaine	20	Case series	Significant pain relief
Schaffer [34]	2015	Acute anterior or global headache	Tx360 device	None	0.5% bupivacaine	93	Randomized placebo-controlled	No statistically significant difference
Sussman et al. [72]	2016	Chronic posttraumatic headache after sport-related concussion	Cotton-tip applicator	None	2% lidocaine and 0.5% bupivacaine	1	Case report	Symptom free at 6-month follow-up

#### Other syndromes

SPG block has been used for a myriad of other conditions not involved in painful syndromes of the head and face. These conditions include seizures associated nasal pathology, arthritic pain and muscle spasm, intercostal neuritis, persistent hiccups, ureteral colic, dysmenorrhea, peripheral painful vascular spasm, complex regional pain syndrome and hypertension (Table 12). Most of these studies reported significant improvement, but none of them had evidence level above case series. There was one randomized-controlled study in assessing the efficacy of SPG block in treating nicotine addiction, but the result was negative [36]. One small double-blind cross-over study examined whether SPG block reduces experimentally induced pain using submaximal effort tourniquet test, but the SPG block failed to make a difference in pain perception [37].

**Table 12:** Studies of SPG blocks for other syndromes

Other syndromes								
Author	Year	Medical problems	Approach	Imaging	Medication	Number of cases	Study design	Outcome
Byrd et al. [73]	1930	“Remote dysfunctions”	Cotton tipped applicator, transnasal approach	None	50% butyn	Over 2000 cases	Case series	Remote dysfunctions were arrested
Sparer et al. [74]	1935	Recurrent convulsive seizures associated with nasal pathology	Needle injection	None	Mixture of alcohol and novocaine	3	Case series	Cessation of seizures
Ruskin et al. [29]	1946	Muscle spasms and arthritic pain	Unknown	None	cocaine, novocaine or nupercaine	68	Case series	Pain partially or completely relieved with SPGB and intramuscular injections of ironyl and calcium ascorbate
Amster et al. [28]	1948	4 migraine, 2 acute torticollis, 12 painful spastic shoulder, 2 intercostal neuritis, 3 herpes zosters, 4 persistent hiccups, 5 ureteral colic, 3 dysmenorrhea, 7 peripheral painful vascular spasm, 61 lumbosacral and sacroiliac pain	Cotton tipped applicator, transnasal approach	None	Nupercaine, pontocaine, monocaine	103	Case series	Relief of pain and spasm in 90% of cases
Ruskin et al. [75]	1949	Arthritic pain	Unknown	None	Unknown	30	Case series	Pain partially or completely relieved with SPGB and iron salt of the adenylic nucleotide
Slade et al. [51]	1986	Tear secretion with topical anesthesia	Needle injection, through the greater palatine foramen	None	2% lidocaine	10	Case-control (using self as control)	Tear secretion significantly reduced by 73% (p < 0.001)
Henneberger et al. [36]	1988	Nicotine addiction	Cotton tipped applicator, transnasal approach	None	Bupivacaine, cocaine or saline	6 with bupivacaine, 5 with cocaine, 6 with saline	Double-blind placebo-controlled	Significantly fewer symptoms of discomfort for patients in the anesthetic treatment groups than the placebo group
Silverman et al. [37]	1993	Experimentally induced pain (submaximal effort tourniquet test)	Cotton tipped applicator	None	20% lidocaine and epinephrine	16 healthy volunteers	Double-blind, cross-over study	No significant difference between experimental and placebo groups
Quevedo et al. [76]	2005	Complex regional pain syndrome involving the lower extremity	Cotton tip applicator, transnasal	None	4% tetracaine	2	Case series	50% pain reduction
Triantafyllidi et al. [77]	2016	Hypertension	Cotton tip applicator, transnasal	None	2% lidocaine	22	Cohort study	Systolic blood pressure significantly decreased by 24 hrs and by 21-30 days

Overall, the grade of recommendation for any of these syndrome remains at C. SPG block is not recommended for nicotine addiction due to the negative randomized study.

#### Summary for SPG block

Grade of recommendation of using SPG block is B for cluster headache, second-division trigeminal neuralgia, migraine, reducing the pain associated with nasal packing removal after nasal operation and for reducing the needs of analgesics after endoscopic sinus surgery. Out of these conditions, SPG block has the best evidence in reducing the needs of analgesics after endoscopic sinus surgery, as there are six randomized-controlled studies. It should be noted that the recommendation for cluster headache, second-division trigeminal neuralgia and migraine are each based on one small study, and it is only meant for acute treatment. There is no positive controlled study warranting chronic treatment with SPG block. For other pain syndromes, grade of recommendations is C due to the lack of positive controlled studies. These syndromes include postdural puncture headache, sphenopalatine maxillary neuralgia, facial neuralgia, sympathetic neuralgia, post-traumatic atypical facial pain, atypical odontalgia, pain from midline granuloma, herpetic keratitis, hemifacial headache, paroxysmal hemicrania, nasal pain, hemicrania continua, trigeminal neuropathy, cancer pain, seizures associated nasal pathology, arthritic pain and muscle spasm, intercostal neuritis, persistent hiccups, ureteral colic, dysmenorrhea, peripheral painful vascular spasm, complex regional pain syndrome and hypertension. Use of SPG block for myofascial pain, including fibromyalgia and head, neck, shoulder myofascial pain and low back pain, is not recommended due to several negative randomized-controlled studies.

### Radiofrequency ablation

Fifteen studies were included on the topic of SPG radiofrequency ablation. One study was a small but positive prospective cohort study for cluster headaches, while the other 14 studies were case reports and case series. There were no controlled studies.

#### Cluster headache

There was one prospective cohort study and eight case reports/series on the treatment of cluster headache. Three case reports were on pulsed radiofrequency and six on continuous radiofrequency ablation (Table 13). Narouze et al. [38] performed a prospective cohort study of 15 cases of chronic cluster headaches treated with radiofrequency ablation using infrazygomatic approach under fluoroscopy guidance. A total of 0.5 mL of lidocaine 2% was injected and 2 radiofrequency lesions were carried out at 80 °C for 60 s each. After the ablation, 0.5 mL of bupivacaine 0.5% and 5 mg of triamcinolone were injected. He reported statistically improved attack intensity, frequency and pain disability index up to 18 months (level 2b). As for side effects: 50% (7/15) reported temporary paresthesias in the upper gums and cheek that lasted for 3-6 weeks with complete resolution. In only one patient, a coin-like area of permanent anesthesia over the cheek persisted. Sanders et al. [39] reported the largest case series of 66 cluster headache patients treated with radiofrequency ablation after 12 to 70 months. He reported complete relief in 60.7% of patients with episodic cluster headache, and in 30% of patients with chronic cluster headache. Of the 66 treated patients, eight patients experienced temporary postoperative epistaxis and 11 patients exhibited cheek hematomas. A partial radiofrequency lesion of the maxillary nerve was inadvertently made in four patients. Nine patients complained of hypoesthesia of the palate, which disappeared in all patients within 3 months.

**Table 13:** Studies of SPG radiofrequency ablation on cluster headache

Cluster headache											
First author	Year	Medical problem	Approach	Imaging	Temperature (°C)	Type of RFA	Parameter	How to identify right spot	Study design	Number of cases	Outcome
Sanders et al. [39]	1997	Cluster headache	Infrazygomatic approach	Fluoroscopy	70	High frequency	50 Hz, 0.2-1 V	Paresthesia in the palate	Case-only	66	60.7% of episodic cluster headache patients received complete relief, 30% in chronic cluster headache patients achieved complete relief
Narouze et al. [38]	2009	Chronic cluster headache	Infrazygomatic approach	Fluoroscopy	80	Unknown	50 Hz at <0.5 V to produce deep paresthesia behind the root of the nose	.	Prospective cohort	15	Mean attack intensity, mean attack frequency, pain disability index significant reduced at 1 year follow-up (P < 0.0005, P < 0.0003, P < 0.002, respectively)
Chua et al. [78]	2011	Cluster headaches	Infrazygomatic approach	Fluoroscopy	42	Pulsed	50 Hz, 0.5-0.7 V	Paresthesia at the root of the nose	Case series	3	Two had excellent relief, one had partial relief by 2 months
Oomen et al. [79]	2012	Atypical facial pain, cluster headache, Sluder’s neuralgia, Sluder’s neuropathy	Infrazygomatic approach	Fluoroscopy	80	Unknown	50 Hz	Paresthesia in the nose and not in the area of the maxillary nerve	Case series	3	Adequate pain reduction: 4/4 in atypical facial pain, 2/3 in cluster headache, 1/2 in Sluder’s neuralgia, 2/2 in Sluder’s neuropathy, 1/1 in posttraumatic neuropathy, 0/1 in post-herpetic neuralgia, 0/1 in SUNCT (60% showed considerable pain relief after a single procedure).
Zarembinski et al. [54]	2014	Drug-resistant chronic cluster headache, with Jacob’s disease	Initially sphenopalatine ganglion block, then radiofrequency.	Fluoroscopy, CT	Unknown	Unknown	Unknown	NA	Case report	1	Pain significantly improved.
Fang et al. [80]	2015	Cluster headache	Infrazygomatic approach	CT	42	Pulsed	Unknown	0.1-0.3 V to induce paresthesia of the nasal root	Case series	16	11 episodic and 1 chronic cluster headache patients had complete relief by 6.3 days. 2 episodic and 2 chronic cluster headache patients had no relief.
Bendersky et al. [81]	2015	Cluster headache	Infrazygomatic approach	Fluoroscopy	42	Pulsed	45 V, 2 Hz, pulse width 20 ms	Paresthesia at the roof of the nose	Case series	3	2 patients had no relief, 1 had relief until 1 month. Continue RFA gave relief to all three patients.
Dharmavaram et al. [82]	2016	Cluster headache	Lateral approach	Fluoroscopy	80	Continuous	Unknown	paresthesia at the root of the nose was obtained at 0.3 V	Case report	1	Pain free for 2 months
Loomba et al. [83]	2016	Cluster headache	Infrazygomatic approach	CT	80	Continuous	50 Hz	<0.3 V to induce paresthesia of the nasal root	Case report	1	Near complete resolution at 6 months

The grade of recommendation is B for treating cluster headache with radiofrequency ablation because of the positive cohort study.

#### Other head and facial pain

There were Seven case reports/series on various head and facial pain other than cluster headaches (all level 4, Table 14). These included Sluder’s neuralgia, posttraumatic headache, chronic head and facial pain, atypical trigeminal neuralgia, atypical facial pain, chronic facial pain secondary to cavernous sinus meningioma, trigeminal neuralgia and SPG neuralgia due to herpes zoster. Akbas et al. [40] reported a 27-case series with various types of head and facial pain. In 35% of the cases, pain was completely relieved, while 42% had moderate relief and 23% had no relief with the SPG radiofrequency ablation. Because there were only case reports and case series available, the grade recommendation is C for any of these conditions.

**Table 14:** Studies of SPG radiofrequency ablation on head and facial pain

First author	Year	Medical problem	Approach	Imaging	Temperature (°C)	Type of RFA	How to identify the right spot	Study design	Number of cases	Outcome
Salar et al. [50]	1987	Sluder’s neuralgia	Lateral extraoral approach	Fluoroscopy	60 and 65	Continuous	0.2-0.3 V, paresthesia in the distribution of the maxillary nerve	Case series	7	Disappearance of the typical pain attacks, lacrimation and nasal secretion, however, a slight, deep-seated troublesome sensation persisted
Shah et al. [84]	2004	Posttraumatic headache	Infrazygomatic approach	Fluoroscopy	42	Pulsed	50 Hz and 0.5 V produced tingling sensation at the root of the nose	Case report	1	Pain reduced from 10/10 to 1/10
Bayer et al. [85]	2005	Chronic head and face pain	Infrazygomatic approach	Fluoroscopy	42	Pulsed	50 Hz up to 1 V, paresthesia elicited at the roof of the nose, motor stimulation performed at 2 Hz to rule out trigeminal contact, which results in rhythmic mandibular contraction	Case series	30	21% had complete pain relief, 65% had moderate pain relief, 14% had no pain relief.
Nguyen et al. [86]	2010	Atypical trigeminal neuralgia	Coronoid approach	Fluoroscopy	42	Pulsed	50 Hz with 1 ms pulse duration, 0.6 V	Case report	1	Symptom-free after 2 yrs.
Oomen et al. [79]	2012	Atypical facial pain, cluster headache, Sluder’s neuralgia, Sluder’s neuropathy	Infrazygomatic approach	Fluoroscopy	80	Unknown	50 Hz, paresthesia in the nose and not in the area of the maxillary nerve	Case series	4 atypical facial pain, 2 Sluder’s neuralgia, 2 Sluder’s neuropathy, 1 post-traumatic neuropathy of infraorbital nerve, 1 postherpetic neuralgia, 1 SUNCT	Adequate pain reduction: 4/4 in atypical facial pain, 2/3 in cluster headache, 1/2 in Sluder’s neuralgia, 2/2 in Sluder’s neuropathy, 1/1 in posttraumatic neuropathy, 0/1 in post-herpetic neuralgia, 0/1 in SUNCT (60% showed considerable pain relief after a single procedure).
Elahi et al. [87]	2014	Facial pain secondary to cavernous sinus meningioma removal	Infrazygomatic approach	Fluoroscopy	80	Continuous	50 Hz, paresthesia in the nasolabial midline region	Case report	1	Satisfactory pain relief at 12 months
Akbas et al. [40]	2016	Atypical facial pain, SPG neuralgia due to herpes zoster, atypical Trigeminal neuralgia	Infrazygomatic approach	Fluoroscopy	42	Continuous	Paresthesia at the roof of the nose at 0.5–0.7 V. To rule out trigeminal contact, motor stimulation at a frequency of 2 Hz was applied	Case series	27	Pain relief not achieved in 23%, completely relieved in 35% and moderately relieved in 42% of patients

#### Summary for SPG radiofrequency ablation

Grade of recommendation is B for applying SPG radiofrequency ablation to intractable cluster headache. The protocol used in the cohort study took infrazygomatic approach under fluoroscopy and two radiofrequency ablations were carried out at 80 °C for 60 s. However, there is not yet a randomized-controlled study to test its efficacy. Grade of recommendation is C for other head and facial pain, including Sluder’s neuralgia, posttraumatic headache, atypical trigeminal neuralgia, atypical facial pain, chronic facial pain secondary to cavernous sinus meningioma, trigeminal neuralgia and SPG neuralgia due to herpes zoster.

### Sphenopalatine ganglion neurostimulation

Eight studies were included for SPG neurostimulation. There was one randomized-controlled study with two long-term follow-ups of the same study and five case report/case series on sphenopalatine ganglion neurostimulation (Table 15).

**Table 15:** Studies of SPG neurostimulation

Neurostimulation										
First author	Year	Medical problem	Stimulator	Approach	Imaging	Types of stimulation	How to identify the right spot	Study design	Number of cases	Outcome
Tepper et al. [45]	2009	Intractable migraine	Medtronic model 3625 or 3628	Infrazygomatic approach	Fluoroscopy	Customized, average amplitude, 1.2 V, pulse rate 67 Hz, pulse width 462 μs	Paresthesia with stimulation at the back of the nose and deep in the back of the soft palate	Case only	11	2 pain-free, 3 had pain reduction, 5 had no response, 1 was not stimulated
Ansarinia et al. [44]	2010	Cluster headache	Medtronic model 3625	Pterygopalatine fossa	Fluoroscopy	Customized, average amplitude, 1.7 V, frequency 88 Hz, pulse width 294 μs	paresthesia with stimulation in the posterior nasopharynx and root of the nose	Case only	6	Total 18 CH attacks, complete resolution with SPG stimulation in 11 attacks, partial in 3, no relief in 4.
Schoenen et al. [41]	2013	Cluster headache	ATI SPG stimulator	Pterygopalatine fossa proximate to the sphenopalatine ganglion	CT	Customized, mean frequency 120.4 Hz, mean pulse width 389.7 μs, mean intensity 1.6 mA	X-ray	Randomized controlled	28 cases, with 3 randomized settings.	Pain relief achieved in 67.1% of full stimulation-treated attacks compared to 7.4% of sham-treated attacks. P < 0.0001
Elahi et al. [47]	2015	Idiopathic right facial pain	Medtronic model 3378	The pterygopalatine fossa	Fluoroscopy	0.5 mV, pulse width 250 – 450 μs, and 40 – 80 Hz	X-ray	Case report	1	2/10 pain on 6-month follow-up
Meng et al. [88]	2016	Cluster headache	Medtronic model 3487A	Pterygopalatine fossa	Fluoroscopy	Bilateral stimulation, right 0-, 1+, 130 Hz, 120 μs, 0.7 V; left 8-, 9+, 130 Hz, 120 μs, 0.8 V	X-ray	Case report	1	Headache frequency reduced to once a week, pain level 1/10 at 4 months
William et al. [46]	2016	Idiopathic facial pain, supraorbital neuropathy, hemicrania continua, facial anesthesia dolorosa, occipital neuropathy	Medtronic Subcompact Octrode	SPG	Fluoroscopy	Unknown	X-ray	Case series	5	80% reported sustained facial pain at mean follow-up of 9.6 months.
Jurgens et al. [42]	2016	Cluster headache	Neurostimulator, described in [41]	Pterylopalatine fossa	CT	Customized, applied as soon as the patient feels cluster headache attacks	X-ray	Cohort study. Long-term follow-up from [41]	33 cases	61% of patients were either acute responder (>50% relief from moderate or greater pain) or frequency responder (>50% in attack frequency) at 24 months
Barloese et al. [43]	2016	Cluster headache	Neurostimulator, described in [41]	Pterylopalatine fossa	CT	Customized, applied as soon as the patient feels cluster headache attacks	X-ray	Cohort study. Long-term follow-up from [41]	33 cases	30% experienced at least 1 episode of complete attack remission (attack-free period exceeding 1 month).

#### Cluster headache

There was one randomized-controlled study with two long-term follow-ups of the same study, and two case reports/series on cluster headache. Schoenen et al. [41] reported a randomized-controlled trial using SPG neurostimulator for patients with refractory cluster headaches. Twenty-eight patients underwent SPG stimulator implantation and stimulations were applied at the onset of cluster headache. The study employed a protocol that randomly inserted a placebo when treatment was initiated by the patient for a cluster headache attack. Three settings were delivered in a randomized fashion (1:1:1): full stimulation (i.e. customized stimulation parameters established during the therapy titration period), sub-perception stimulation, and sham stimulation. A total of 566 cluster headaches were treated, and pain relief was achieved in 67.1% of patients receiving full stimulation compared to 7.4% receiving sham treatment (*P* < 0.0001). Pain relief using sub-perception stimulation was not significantly different from sham stimulation (*P* = 0.96). Acute rescue medication was used in 31% of cluster headache attacks in patients receiving full stimulation, compared to 77.4% treated with sham stimulation (P < 0.0001) and 78.4% with sub-perception stimulation (P < 0.0001). In terms of side effect, most patients (81%) experienced transient, mild to moderate loss of sensation within distinct maxillary nerve regions; 65% of events resolved within 3 months. Jurgens et al. [42] reported a cohort study from the subjects who volunteered to be followed for 24 months from the study by Schoenen et al. In this study, 61% of patients were either acute responder (>50% relief from moderate or greater pain) or frequency responder (>50% in attack frequency) at 24 months. Barloese et al. [9] analyzed participants who experienced remission from the same dataset. 30% of participants were found to have at least 1 episode of complete attack remission in the 24-month period. Ansarinia et al. [44] reported a case series of 6 patients. Out of the 18 attacks recorded, there were 11 attacks receiving complete relief from the stimulations, 3 getting partial relief and 4 without relief.

With the positive randomized-controlled trial, the grade of recommendation is B for using SPG neurostimulation on cluster headache. Given the positive effect from these studies, further trials are encouraged.

#### Migraine headache

There was one case series of 11 cases on SPG neurostimulation in acutely treating intractable migraine headaches [45]. In this study, 11 patients with a history of migraine headache for a mean of 20 years were studied. Spontaneous and induced migraine headaches were acutely treated with SPG neurostimulation. Out of the 11 treated, two patients were pain-free, three had some pain reduction, while five had no response. Because of the largely negative response, there is currently not enough evidence for treating intractable migraine with SPG neurostimulation.

#### Other head and facial pain

There was one case series and one case report on other types of head and facial pain. William et al. [46] reported a case series on idiopathic facial pain, supraorbital neuropathy, hemicrania continua, facial anesthesia dolorosa and occipital neuropathy. SPG neurostimulation was combined with trigeminal or peripheral stimulation. 80% of the patients reviewed reported sustained relief in facial pain. It is unclear whether SPG stimulation alone would provide the same relief in these cases. Elahi et al. [47] reported a single case of SPG neurostimulation for idiopathic facial pain with good success.

Given the sparse literature, the grade of recommendation is C for SPG neurostimulation in idiopathic facial pain and D for SPG stimulation combined with trigeminal/peripheral stimulation in supraorbital neuropathy, hemicrania continua, facial anesthesia dolorosa and occipital neuropathy.

#### Summary for SPG neurostimulation

Grade of recommendation is B for applying SPG neurostimulation to cluster headache and C for idiopathic facial pain. There may be a role of combined SPG and trigeminal or peripheral neurostimulation in isolated cases. Due to its invasive nature, SPG neurostimulation warrants further investigations with more high quality, large-scale studies.

## Discussion

### Sphenopalatine ganglion block

Sphenopalatine ganglion block has been used for over a century. In 1908, Sluder first proposed that inflammation in the posterior ethmoid and sphenoid sinuses may be involved in unilateral facial pain associated with tearing, congestion and rhinorrhea. He also claimed to have successfully treated facial neuralgia, asthma, earache and lower-half headache. Over time, the term Sluder’s neuralgia has varied definitions across the medical literature. Its characteristics mostly resemble cluster headache and it has been suggested that the term Sluder’s neuralgia be discarded [48]. However, an analysis suggested that cluster headache and Sluder’s neuralgia may be two different entities [49]. This review kept Sluder’s neuralgia and cluster headaches as two distinct type of headaches because of the differences. Since Sluder’s first publication, SPG block has been reported to be used successfully in treating multiple pain syndromes, including cluster headaches, trigeminal neuralgia, migraine, postherpetic neuralgia and atypical facial pain. It was also used for treating intractable cancer pain of the head and face as well as facial pain management after endoscopic sinus surgery. However, for most pain syndromes the evidence for using SPG nerve block remains at case report and case series level. There were a few small yet positive randomized-controlled studies in nitroglycerin-induced cluster headache, second-division trigeminal neuralgia, migraine, reducing the pain associated with nasal packing removal after nasal operation and for reducing the needs of analgesics after endoscopic sinus surgery. It should be emphasized that the evidence for treating these conditions with SPG block is based on very few small studies. The exception lies in reducing the needs of analgesics after endoscopic sinus surgery, which is backed by five randomized-controlled studies. It should be also noted that long-term treatment may not be beneficial, as demonstrated by the chronic repetitive block study in migraine by Cady et al. [16]. When SPG block is offered as a treatment option, patients should be informed of such caveats.

#### Blocking strategies

Several techniques exist for SPG blockade. Four types of applications exist: cotton-tip applicator, Tx360 device, nasal spray and needle injections. Three main types of approaches exist: transnasal, transoral and infrazygomatic approaches. Cotton-tip applicator, Tx360 device and nasal spray can only be applied through the transnasal approach. Needle injection, on the other hand, can be performed in any approach. Applied local anesthetics included lidocaine, bupivacaine, ropivacaine, levobupivacine, mepivacaine, novocaine, nupercaine, pontocaine, monocaine, tetracaine, and prilocaine, with varying concentrations, but lidocaine and bupivacaine were by far the most common. Other medications include cocaine, ethanol and phenol. Co-medications included epinephrine, triamcinolone and dexamethasone. Some studies used fluoroscopy or CT to guide needle placement. Unfortunately, there are no head-to-head trials comparing the efficacy among different blocking strategies. The recommendations made in this article are based on strategies used in the positive controlled studies.

#### Side effects

Side effects from SPG blockade is typically local. Potential side effects are numbness and stinging at the root of the nose and palate, numbness or lacrimation of ipsilateral eye, and bitter taste and numbness of the throat. With needle injection techniques, there is also the risk of bleeding, infection and epistaxis.

### Sphenopalatine ganglion radiofrequency ablation

The use of radiofrequency on sphenopalatine ganglion was first reported by Salar et al. [50] for treating Sluder’s neuralgia. Since the first report, there were multiple case reports on using SPG radiofrequency ablation in treating head and facial pain. About half of the reports focused on treating cluster headaches, but it has also been successfully used on patients with post-traumatic headache, atypical trigeminal neuralgia and anesthesia dolorosa after cavernous meningioma surgery. However, most of the literature today remains at the case report and case series level. There was only one small prospective cohort study on the effectiveness of SPG radiofrequency ablation. Well-controlled studies are yet to be performed to confirm the validity of this therapeutic modality in treating headache and facial pain.

Compared to the short-lived effect of SPG block, SPG radiofrequency ablation tend to be long lasting. Narouze et al. [38] reported statistically improved attack intensity, frequency and pain disability index up to 18 months in patients who underwent SPG radiofrequency ablation. As a comparison, Costa et al. [6] only reported shorter cluster headache duration with SPG block, and Cady et al. reported only up to 24 h of relief in chronic migraine [15] while no difference was found at 1 and 6 months with repetitive SPG block [16].

#### Ablation strategies

Most radiofrequency ablation of SPG were carried out with the infrazygomatic approach. The most commonly used temperature is 80 °C for thermal ablation, and 42 °C for pulsed ablation. There is unfortunately no head-to-head comparison between the two types of ablations. All studies confirmed the position of RF cannula/probe by applying low voltage sensory stimulation (between 0.2-0.1 V) while patients felt paresthesia or tingling sensation at the root of the nose. The only study with evidence level above case series was a cohort study on patients with chronic cluster headache [38]. In this positive study, the authors applied 2 rounds of thermal ablation at 80 °C for 60 s each. Pre- and post-ablation medications were also given (pre: 0.5 ml of 2% lidocaine; post: 0.5 ml of 0.5% bupivacaine and 5 mg of triamcinolone).

#### Side effects

Based on the study by Narouze et al. [38], about 50% (7/15) reported temporary paresthesias in the upper gums and cheek that lasted for 3-6 weeks with complete resolution. Rare permanent small zone of hypoesthesia over the cheek could also happen. In the large case series by Sanders et al. [39], of the 66 treated patients, eight patients experienced temporary postoperative epistaxis and 11 patients exhibited cheek hematomas. A partial radiofrequency lesion of the maxillary nerve was inadvertently made in four patients. Nine patients complained of hypoesthesia of the palate, which disappeared in all patients within 3 months.

### Sphenopalatine ganglion neurostimulation

Neurostimulation has emerged in recent years as a potential therapeutic modality for headaches and facial pain. Even though number of studies on SPG neurostimulation has not been abundant, the overall quality of the studies has been high. The study by Shoenen et al. [41] was the only randomized-controlled study in using SPG neurostimulation to treat chronic cluster headache. Despite the small number of participants, the effectiveness is demonstrated by the large effect size and highly significant *P* value. The two long-term follow-up articles continued to support the effectiveness of such intervention [42, 43]. These three studies combined is the strongest piece evidence to date, suggesting that SPG neurostimulation is effective in treating cluster headache. There were other isolated case reports on the successful application of SPG neurostimulation to other pain syndromes, but higher level of evidence is lacking.

#### Stimulation strategies

Stimulation settings vary widely across study subjects, stimulator models and studies. In the controlled study by Schoenen et al. [41], the mean frequency was 120.4 ± 15.5 Hz, mean pulse width 389.7 ± 75.4 μs with mean intensity 1.6 ± 0.8 mA during full stimulation. These numbers are for references only, and the stimulation setting should be individualized based on responses.

#### Side effects

In Schoenen’s controlled study [41], the most common acute side effects are sensory disturbances (81%), pain (38%), swelling (22%). Other side effects included tooth pain (16%), trismus (16%), headache (9%), dry eye (9%), and hematoma (9%). Across all 32 patients, five device- or procedure-related serious adverse events occurred. The most common serious adverse events are due to erroneous lead placements and lead migration to adjacent nerves.

### Limitations

There are several limitations in our review. Firstly, articles could have been missed because only Pubmed, CENTRAL and Google Scholar were used. Second, most of the studies included in this review were case studies and case reports. By nature of these kinds of studies, publication bias will be skewed toward positive outcomes. Thirdly, due to the paucity of controlled studies, meta-analysis could not be adequately performed to create a quantitative analysis. Despite these limitations, this study was the first to systematically summarize SPG interventions. As more controlled studies become available, meta-analysis will be possible and thus providing better level of evidence in this developing field.

## Conclusions

SPG has been the target for treating pain syndrome in the head and face for over a hundred years. The strongest evidence lies in using SPG block, radiofrequency ablation and neurostimulation on cluster headache. Sphenopalatine ganglion block also has good evidence in treating trigeminal neuralgia, migraines, reducing the needs of analgesics after endoscopic sinus surgery and reducing pain associated with nasal packing removal after nasal operations. Large-scale, double-blinded, randomized-controlled studies are warranted in establishing these techniques in treating cluster headache and other head and facial pain.

## Additional file

Additional file 1: PRISMA checklist. (DOC 64 kb)

## Abbreviations

SPG: Sphenopalatine ganglion
TAC: Trigeminal autonomic cephalalgia
